# Biochemical characterization of a recombinant Japanese encephalitis virus RNA-dependent RNA polymerase

**DOI:** 10.1186/1471-2199-8-59

**Published:** 2007-07-11

**Authors:** Yeon-Gu Kim, Ji-Seung Yoo, Jung-Hee Kim, Chan-Mi Kim, Jong-Won Oh

**Affiliations:** 1 Department of Biotechnology, Yonsei University, 134 Shinchon-dong, Seodaemun-gu, Seoul 120-749, Korea

## Abstract

**Background:**

Japanese encephalitis virus (JEV) NS5 is a viral nonstructural protein that carries both methyltransferase and RNA-dependent RNA polymerase (RdRp) domains. It is a key component of the viral RNA replicase complex that presumably includes other viral nonstructural and cellular proteins. The biochemical properties of JEV NS5 have not been characterized due to the lack of a robust *in vitro *RdRp assay system, and the molecular mechanisms for the initiation of RNA synthesis by JEV NS5 remain to be elucidated.

**Results:**

To characterize the biochemical properties of JEV RdRp, we expressed in *Escherichia coli *and purified an enzymatically active full-length recombinant JEV NS5 protein with a hexahistidine tag at the N-terminus. The purified NS5 protein, but not the mutant NS5 protein with an Ala substitution at the first Asp of the RdRp-conserved GDD motif, exhibited template- and primer-dependent RNA synthesis activity using a poly(A) RNA template. The NS5 protein was able to use both plus- and minus-strand 3'-untranslated regions of the JEV genome as templates in the absence of a primer, with the latter RNA being a better template. Analysis of the RNA synthesis initiation site using the 3'-end 83 nucleotides of the JEV genome as a minimal RNA template revealed that the NS5 protein specifically initiates RNA synthesis from an internal site, U_81_, at the two nucleotides upstream of the 3'-end of the template.

**Conclusion:**

As a first step toward the understanding of the molecular mechanisms for JEV RNA replication and ultimately for the *in vitro *reconstitution of viral RNA replicase complex, we for the first time established an *in vitro *JEV RdRp assay system with a functional full-length recombinant JEV NS5 protein and characterized the mechanisms of RNA synthesis from nonviral and viral RNA templates. The full-length recombinant JEV NS5 will be useful for the elucidation of the structure-function relationship of this enzyme and for the development of anti-JEV agents.

## Background

Japanese encephalitis virus (JEV) is the most common cause of epidemic viral encephalitis worldwide, with approximately 50,000 cases and 15,000 deaths annually throughout a wide geographical range [1]. Since the prototype Nakayama strain of JEV was first isolated in 1935, epidemics and sporadic cases of Japanese encephalitis have occurred in temperate and tropical zones of Asia as well as in non-Asian regions, including Cambodia, China, Indonesia, India, Japan, Malaysia, Myanmar, Nepar, Sri Lanka, Thailand, Vietnam, the south eastern Russian federation, and Australia [2,3].

JEV is a member of *Flaviviridae *family, which consists of the genera *Flavivirus *(JEV, dengue virus [DEN], yellow fever virus [YF], West Nile virus [WNV], Kunjin virus [KUN], Murray Valley encephalitis virus), *Pestivirus *(bovine viral diarrhea virus [BVDV], Classical swine fever virus [CSFV]), and *Hepacivirus *(hepatitis C virus, [HCV]). JEV is an enveloped, positive-stranded RNA virus whose genome consists of a single-stranded RNA molecule of approximately 11 kb. The RNA genome of JEV consists of 98-nucleotide (nt) long 5' untranslated region (UTR) with the type-1 cap structure at its 5' terminus, a single open reading frame (ORF), and a 585-nt long 3'-UTR with no poly(A) tail at its 3' terminus [4]. The single large ORF encodes a polyprotein of ~3,400 amino acids that is subsequently processed by both host and viral proteases into three structural proteins and seven nonstructural proteins [4]. The structural proteins (capsid, membrane, and envelope proteins) are contained in the N-terminal third of the polyprotein, while the nonstructural proteins (NS1, NS2A, NS2B, NS3, NS4A, NS4B and NS5) are located in the C-terminal two-thirds of the polyprotein. NS5 is the largest nonstructural protein of JEV. Analysis of the amino acid sequence of NS5 led to the prediction that it carries both methyltransferase and RNA-dependent RNA polymerase (RdRp) activities. The 5' RNA methyltransferase activity should be located on the N-terminal portion because of the presence of conserved motifs found in other viral methyltransferases [5-7]. The C-terminal region of NS5, which contains the conserved RdRp motifs [8,9] and the Gly-Asp-Asp (GDD) motif found in the active site of many viral RdRps [10], is likely responsible for the RNA polymerase activity.

RNA synthesis by various RNA virus RdRps has been shown to occur by either a *de novo *initiation mechanism in which first nucleotide serves as a primer to provide the 3'-hydroxyl group, or via a primer-(an oligonucleotide, a protein linked to nucleotides, or intramolecular self priming) dependent mechanism [11-16]. *De novo *initiation results in the synthesis of various RdRp products including a template-size product initiated from the most 3'-end or an RNA molecule smaller than the template (via internal initiation) [11,15,17]. Although *de novo *initiation has been demonstrated for a number of *Flaviviridae *RdRps, including those from WNV, DEN, KUN, BVDV, CSFV, and HCV [15,17-22], the biochemical properties JEV RdRp and its RNA synthesis initiation mechanism have not been yet analyzed in detail due to the lack of an *in vitro *RdRp assay system. In this work, we expressed and purified JEV NS5 protein from *Escherichia coli *and characterized its biochemical properties and *in vitro *RNA synthesis initiation mechanism.

## Results

### Expression and purification of recombinant JEV NS5 protein

**Figure 1:**
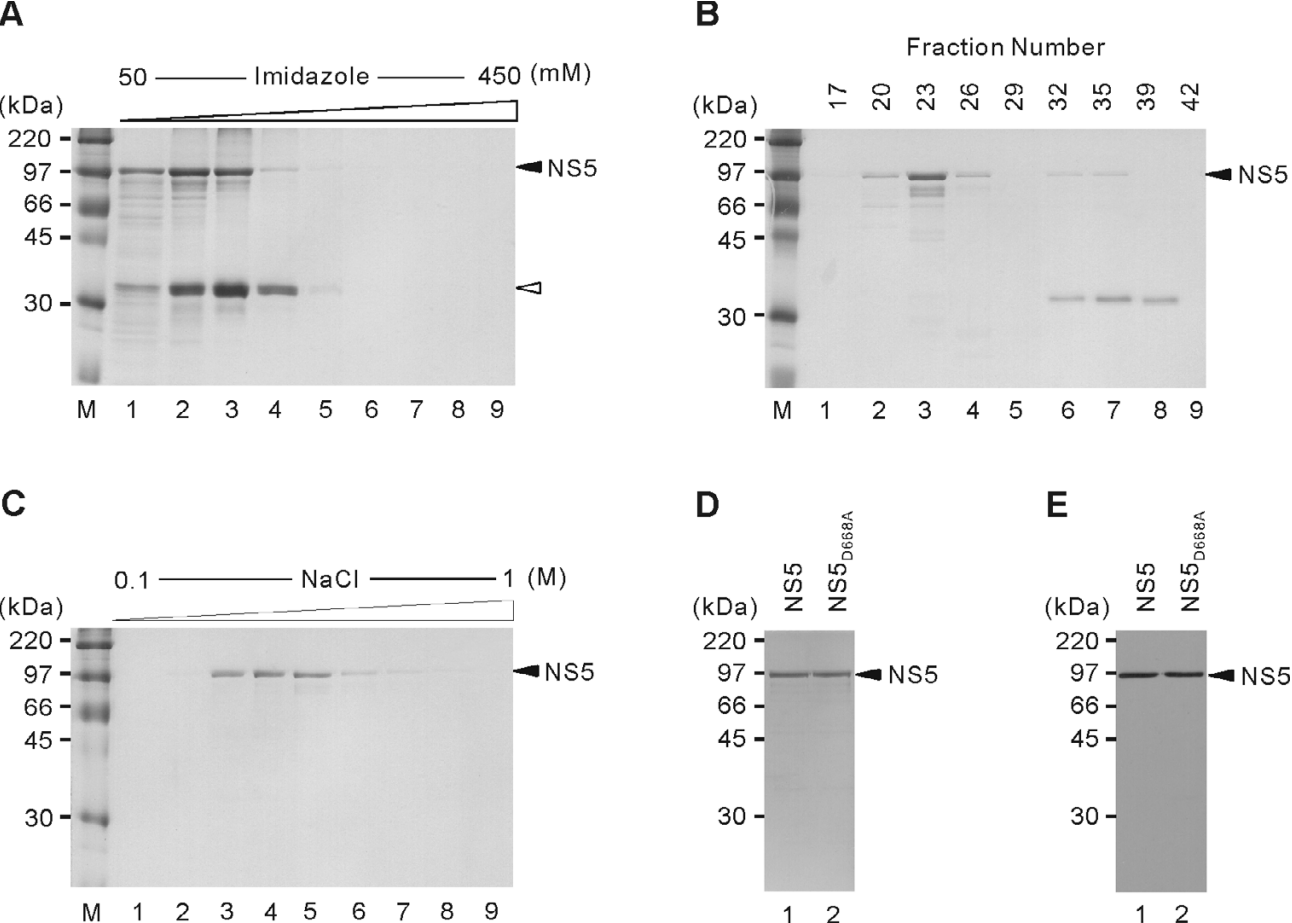
**Purification of recombinant JEV NS5 protein**. JEV NS5 protein was expressed in *E. coli *and purified by Ni-NTA chromatography, gel filtration chromatography, and ion-exchange chromatography using an SP-Sepharose column. (A) Imidazole elution profile of JEV NS5 from Ni-NTA resin. (B) Gel filtration chromatography elution profile of JEV NS5. (C) NaCl-elution profile of JEV NS5 from an SP-Sepharose column. (A-C) Fractions from each purification step were resolved by SDS-12% PAGE and stained with Coomassie brilliant blue. (D) JEV NS5 and its mutant NS5_D668A _from a peak fraction eluted from an SP-Sepharose column were resolved by SDS-12% PAGE and visualized by silver staining. (E) Western blot analysis of the purified JEV NS5 and NS5_D668A _using an anti-His_6 _antibody. The sizes of protein markers are indicated in kilodaltons. Closed and open arrowheads indicate the full-length JEV NS5, and its major cleaved form identified by MALDI-TOF analysis, respectively.

To investigate the biochemical properties of JEV RdRp, recombinant JEV NS5 protein with a hexahistidine tag at the N-terminus was expressed in *E. coli*. The *E. coli *cells transformed with an expression vector containing JEV NS5-coding gene were induced with IPTG at low temperature (18°C) to obtain soluble NS5 protein. Western blot analysis of the soluble fraction and total lysates with an anti-His antibody detected ~105-kDa intact JEV NS5 proteins along with ~35-kDa cleaved form of the NS5 (data not shown). These two proteins were co-purified by nickel affinity chromatography (Fig. 1A). The proteins were excised from the gel and in-gel digested with trypsin. The resulting peptide mixtures were analyzed by MALDI-TOF mass spectrometry. Both of these proteins were identified as JEV NS5 protein (data not shown). The 35-kDa C-terminally truncated NS5 proteins were successfully removed by subsequent gel filtration chromatography (GFC) (Fig. 1B, lanes 2–4). The GFC fractions containing the intact form of NS5 were further purified by SP-Sepharose cation exchange chromatography (Fig. 1C). The JEV NS5 mutant protein NS5_D668A _was also similarly purified. The eluate from the SP-Sepharose column contained near homogenous NS5 proteins, as shown by silver staining (Fig. 1D) and Western blot analysis (Fig. 1E).

### Analysis of the RdRp activity of JEV NS5

**Figure 2:**
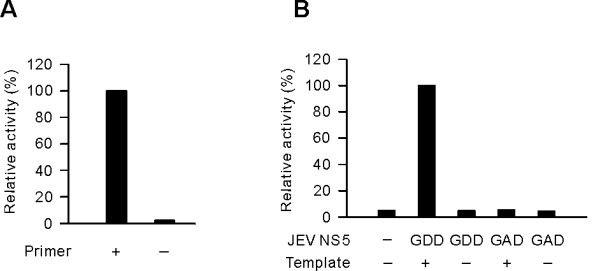
**RdRp assay using a poly(A) template and oligo(U)_20 _primer**. (A) Primer-dependent RNA synthesis. RdRp assays were performed with the purified JEV NS5 using a poly(A) RNA template in the presence (+) or absence (-) of the primer oligo(U)_20_. (B) RdRp assays were performed with the purified wild-type NS5 (GDD) and its mutant NS5_D668A _(GAD) in the presence (+) or absence (-) of a poly(A) RNA template. Relative RdRp activities (%), which were obtained by comparing the ^32^P-UMP incorporation measured by liquid scintillation counting with that obtained for the reaction with the template and primer, 3.0 × 10^5 ^cpm, are presented.

To test the RdRp activity of the purified recombinant JEV NS5 protein, we first performed an RNA polymerase assay with poly(A) RNA template in the absence and presence of primer oligo(U)_20_. The RdRp activity assay conditions used initially were similar to those for HCV NS5B RdRp [12], except that 2.5 mM of both Mn^2+ ^and Mg^2+ ^were added. The RNA polymerase activity of JEV NS5 was only observed in the presence of primer (Fig. 2A). The GDD motif conserved in most RdRps of plus-strand RNA viruses is important for metal binding and is considered to be the catalytic site of the enzyme [8]. Mutation of the first Asp in the GDD motif to Ala in NS5_D668A _resulted in loss of the RdRp activity (Fig. 2B). These results demonstrated that the recombinant JEV NS5 RdRp alone is capable of synthesizing RNA *in vitro *without the help of other viral and/or cellular protein(s).

**Figure 3:**
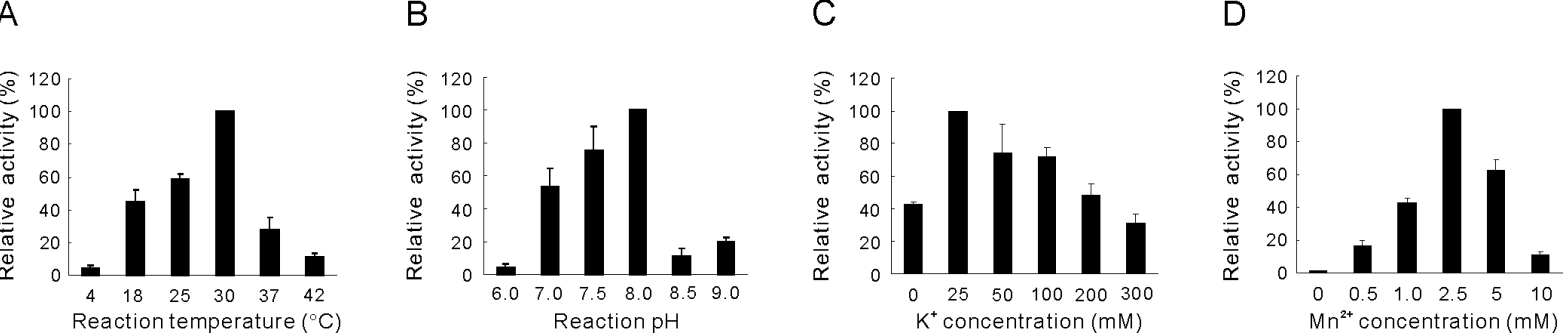
**Optimization of JEV RdRp assay conditions**. Effect of temperature (A), pH (B), K^+ ^ion (C), and Mn^2+ ^ion (D) on JEV RdRp activity. RdRp assays were performed with the poly(A)/(U)_20 _template under the indicated conditions. The RdRp activity was measured as in Figure 2 and is presented as the percentage of that observed under each optimal condition. Shown is the mean and standard error from three independent experiments.

**Figure 4:**
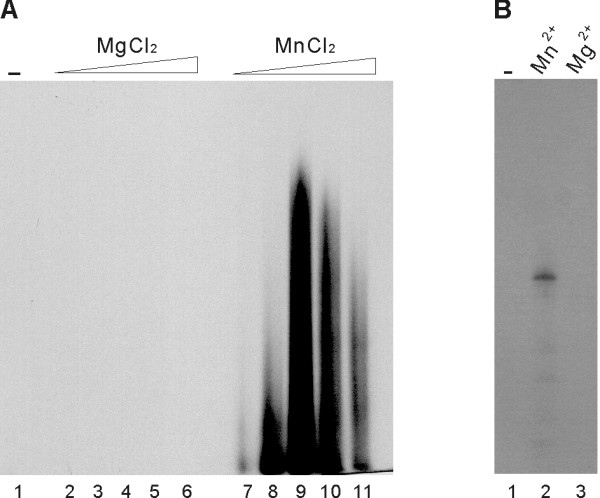
**Dependence of JEV RdRp activity on Mn^2+^ion**. (A) RdRp assays were performed with the poly(A)/(U)_20 _template (A) in the absence (lane 1) or in the presence of increasing concentrations of MgCl_2 _or MnCl_2 _(lanes 2–6 and 7–11; 0.5, 1.0, 2.5, 5.0, and 10 mM of MgCl_2 _and MnCl_2_, respectively). (B) RdRp assays were performed with the 83-nt RNA representing the 3' end of the plus-strand JEV genome, in the absence of metal ions (lane 1) or in the presence of 2.5 mM of the divalent metal ion indicated above the autoradiogram (lanes 2 and 3). RdRp products were denatured and resolved on a medium size (20 × 20 cm) denaturing 5% polyacrylamide gel, and subjected to autoradiography.

Having identified the RNA polymerase activity of the purified NS5 protein, we optimized the RdRp assay conditions by using the poly(A) RNA/oligo(U)_20 _as a template. The optimal temperature for maximal activity was determined to be 30°C (Fig. 3A). The RdRp activity of JEV NS5 was found to be optimal at pH of 8.0; rapid decrease of activity was observed above pH 8 (Fig. 3B). Potassium (K^+^) ion exhibited a stimulatory effect at 25 mM, while the stimulatory effect decreased when the concentration of K^+ ^was higher than 25 mM (Fig. 3C). All known DNA and RNA polymerases require a divalent cation cofactor for optimal activity [8]. Divalent metal ions are also essential cofactors for viral RdRps [16]. The enzymatic activity of JEV NS5 was dependent on Mn^2+ ^with the optimal concentration being 2.5 mM (Fig. 3D). The JEV NS5 exhibited a strong preference for Mn^2+ ^over Mg^2+ ^for RNA synthesis using the poly(A)/U_20 _substrate (Fig. 4A). No detectable level of RdRp activity was observed in the presence of the same range of Mg^2+ ^concentration as that used for Mn^2+ ^(0.5 to 10 mM). Similarly, complete dependence on Mn^2+ ^was observed with a JEV genome-derived RNA template, the 83-nt RNA from the 3'-end of JEV genome (Fig. 4B).

### *De novo *initiation of RNA synthesis from the plus- and minus-strand 3'-UTR of JEV genome

**Figure 5:**
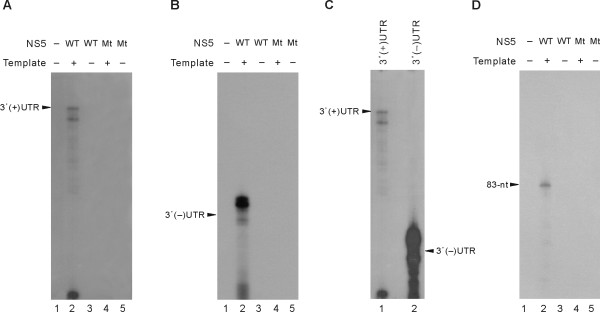
***De novo *initiation of RNA synthesis from the plus- and minus-strand 3'-UTR of the JEV genome**. RdRp assays were performed with the purified wild-type NS5 (WT) and mutant NS5_D668A _(Mt) in the presence (+) or absence (-) of JEV 3'(+)UTR RNA (A and C), 3' (-)UTR RNA (B and C), and the 83-nt RNA (D) template. RdRp products were analyzed as in Figure 4 by autoradiography. Arrowheads indicate the template positions visualized by ethidium-bromide staining of the gels.

*Cis*-acting RNA elements required for viral RNA replication by various viral RdRps are known to reside at the 3'-end of both plus- and minus-strand viral genomes, thereby allowing initiation of RNA synthesis of both polarities. To investigate the RNA synthesis initiation mechanism with the purified recombinant JEV NS5, we performed RdRp assays with JEV genome-derived RNA templates, 3'(+)UTR and 3'(-)UTR RNA, that represent the 3'-end of JEV plus- and minus-strand RNA, respectively. The RNA templates were synthesized by *in vitro *transcription using T7 RNA polymerase and PCR-amplified DNA templates under the control of T7 RNA polymerase promoter. The recombinant JEV NS5 was able to use both JEV 3'(+)UTR and 3'(-)UTR RNA as templates (Figs. 5A–C). RNA synthesis from these templates did not require an exogenous primer, indicating that JEV NS5 can start RNA synthesis *de novo *using a nucleotide as a primer. The major RNA product synthesized from the 3' (+)UTR co-migrated with the template (Fig. 5A, lane 2). In contrast, the major RNA products synthesized from the 3'(-)UTR RNA template migrated slightly slower than the template RNA (Fig. 5B, lane 2). Moreover, through comparison of RNA product amounts synthesized from the 3'(+)UTR and 3'(-)UTR RNA template (Fig. 5C), we found that JEV NS5 apparently recognized a *cis*-acting element in the 3'(-)UTR more efficiently than the one in the 3'(+)UTR for the synthesis of plus-strand JEV RNA. These results indicated that JEV NS5 can synthesize RNA *de novo *using JEV genome-derived RNA templates. The lack of labeled products without an RNA template (Figs. 5A, B, and 5D, lane 3) indicated that the JEV NS5 preparation was not contaminated with any RNAs that could serve as a template. In addition, no RNA products were synthesized by the JEV NS5 mutant NS5_D668A_, indicating that the NS5 protein was not associated with enzymes that can label exogenous RNA templates (Figs. 5A, B, and 5D, lanes 4 and 5).

**Figure 6:**
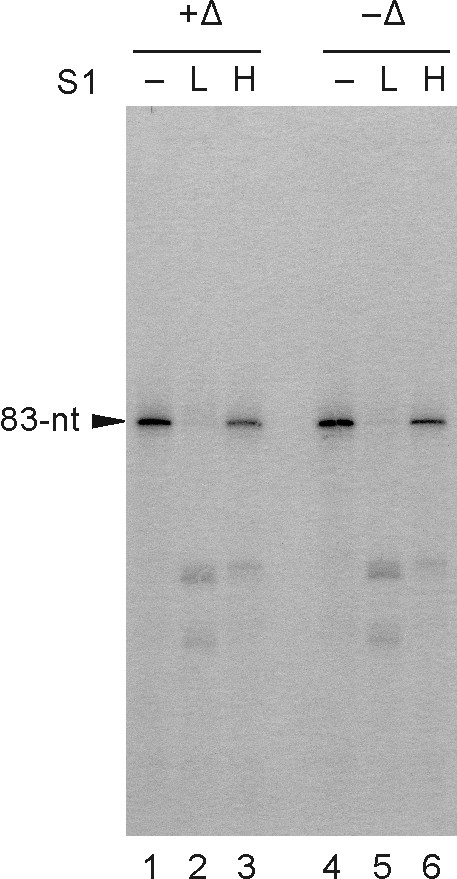
**Nuclease S1 treatment of the RNA products synthesized from the 83-nt RNA template**. Heat-denatured (+Δ) or untreated (-Δ) RdRp products synthesized from the 83-nt RNA, which represents the 3'-terminal region of JEV genome, were left untreated (-) or digested with nuclease S1 (S1), and resolved on a denaturing polyacrylamide gel. Nuclease S1 treatments were performed in 50 mM NaCl (L; low salt) or 500 mM NaCl (H; high salt). Arrowhead indicates the position of the 83-nt RNA template.

In a previous report, JEV NS5 was shown to bind to the 83-nt stem-loop structure formed with the 3'-terminal region of JEV genome, as demonstrated by UV-cross-linking experiments using JEV-infected cell extracts [23]. We also were able to confirm the direct interaction between the purified NS5 and this *cis*-acting RNA element by electrophoretic mobility shift assay (data not shown). These results prompted us to test whether JEV NS5 can utilize this 83-nt RNA as a template for minus-strand RNA synthesis. As expected by its binding ability to the 83-nt RNA, NS5 could direct RNA synthesis to yield a major RdRp product that migrated similarly to the template RNA on a denaturing polyacrylamide gel (20 × 20 cm) (Fig. 5D). There was no generation of RNA products bigger than the template, suggesting that NS5 does not have a detectable level of TNTase activity that would add nucleotide(s) to the 3'-end of the template, under the standard RdRp reaction conditions we used. Furthermore, we observed no dimer-size products, suggesting there was no "copy-back" initiation mediated by intramolecular priming. Next, we analyzed whether the products synthesized from this minimal RNA template are in the single- or double-stranded form. We treated the RNA products with nuclease S1 under low or high salt conditions and found that they were sensitive to nuclease S1 digestion regardless of heat denaturation prior to the nuclease treatment under the low salt (50 mM NaCl) condition (Fig. 6, lanes 2 and 5). In contrast, the products were almost resistant to nuclease S1 under the high salt (500 mM NaCl) condition (lanes 3 and 6), which is a favorable condition for forming a stable RNA duplex. This result suggested that the most of RdRp products directed by the 83-nt RNA template might form a stable RNA duplex with the template or with the product itself.

### *De novo *internal initiation of RNA synthesis from the 83-nt RNA template

**Figure 7:**
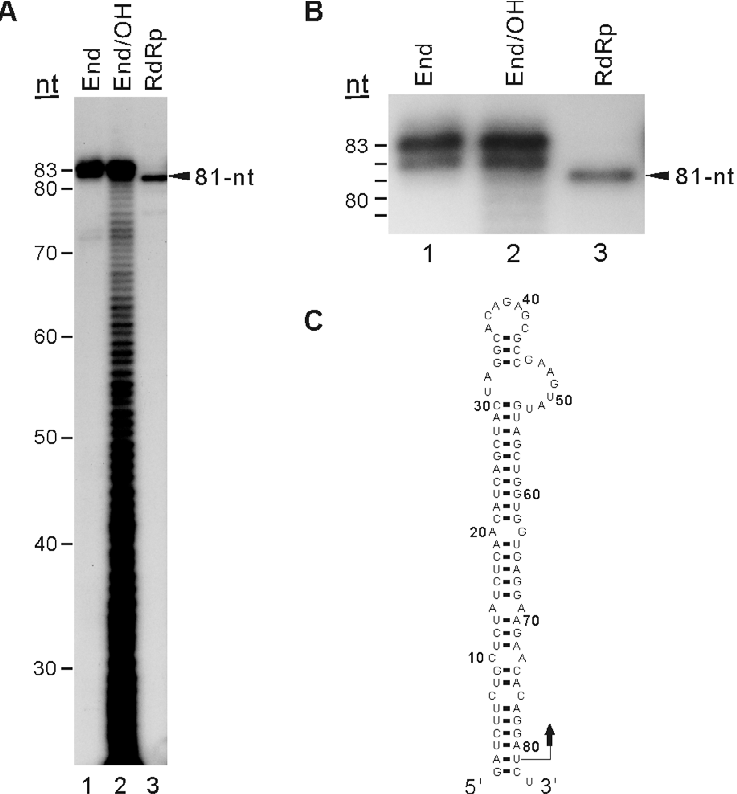
**Mapping of the RNA synthesis initiation site on the 83-nt RNA template**. The RdRp assay was performed with the 83-nt RNA template. (A) An autoradiogram showing the major RNA product synthesized by JEV NS5 using the 83-nt RNA template. Products were resolved on a 5% polyacrylamide sequencing gel (20 × 40 cm) containing 8 M urea. The RNA size markers, 5'-end labeled RNA template (End), and a set of labeled RNA fragments generated by alkaline hydrolysis of the 5'-end labeled RNA template (End/OH), were resolved on the same gel. Arrowhead indicates the internally initiated 81-nt RNA product. (B) The close-up autoradiogram of the same gel shown in (A). (C) Secondary structure of the 83-nt RNA template predicted by the Mfold program. Bent arrow denotes the predicted RNA synthesis initiation site.

**Figure 8:**
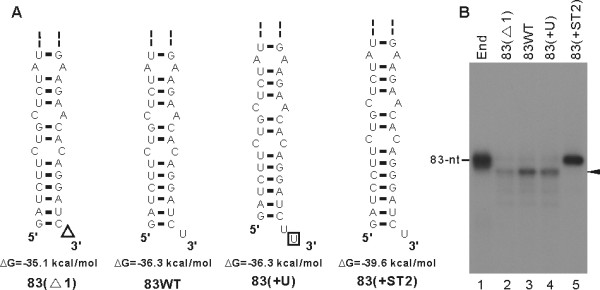
**Effect of nucleotide addition and deletion at the lower part of the stem-loop structure of the 83-nt RNA template on RNA synthesis initiation**. (A) Sequences and secondary structures of wild-type 83-nt RNA and its derivatives. Names and ρ G are shown below the structures predicted by the Mfold program. Partial structures representing the lower stem-region are shown. The deleted and added nucleotides are indicated by ρ and boxed, respectively. (B) RNA synthesis directed by the wild-type 83-nt RNA and its derivatives. RdRp assays were performed with the RNA templates indicated above the autoradiogram, and products were resolved a 5% polyacrylamide sequencing gel (20 × 40 cm) containing 8 M urea. Arrowhead indicates the 81-nt internally initiated RNA product. Representative data from three independent experiments are shown.

To fine map the initiation site of RNA synthesis from the 83-nt RNA template, we resolved the RdRp-reaction product on a denaturing polyacrylamide sequencing gel (20 × 45 cm) and determined the exact size of the RNA product by running in parallel with RNA size markers. The result showed that the size of the major product from the 83-nt RNA template is 81-nt long, shorter than the template RNA (Figs. 7A and 7B, lane 3), indicating that JEV NS5 initiated RNA synthesis from the internal nucleotide U_81 _of the template, as depicted in Fig. 7C. We attempted, without success, to detect 5'-end labeled internally initiated products from the RdRp assays performed in the presence of [γ-^32^P] ATP and all four cold ribonucleotides (data not shown). The limited amount of [γ-^32^P] ATP (0.33 μM) and cold ATP (1 or 5 μM), which might be lower than the K_*m *_value for ATP required for efficient initiation of RNA synthesis, and less sensitive 5'-end labeling of the internally initiated RNA products with single radiolabeled ribonucleotide than the internal labeling at multiple sites with [α-^32^P] UTP, may explain this failure. Therefore, instead of using the [γ-^32^P]-ATP incorporation assay, we performed RdRp assays with insertion and deletion mutants of the 83-nt RNA, namely 83(Δ1) (deletion of U residue from the 3'-end of the 83-nt RNA), 83(+U) (addition of extra U residue to the 3'-end of the 83-nt RNA), and 83(+ST2) (addition of single base pair at the lower stem-region of the 83-nt RNA) (Fig. 8A) to confirm the internal *de novo *initiation of RNA synthesis. Strikingly, the first two derivatives, 83(Δ1) and 83(+U), still directed the synthesis of an 81-nt RNA product co-migrating with the RNA product synthesized from the wild-type template (Fig. 8B, compare lanes 2 and 4 with lane 3). Deletion of one nucleotide from the 3'-terminal end of the template or addition of one nucleotide to the 3'-end did not alter the size of the reaction product, indicating that RNA synthesis initiation did not start from the 3'-end of the template. In contrast, the 83(+ST2) RNA (total 85-nt in length) with an additional base pair at the lower part of the stem structure yielded an 83-nt product (lane 5). These results clearly indicated that the NS5 initiates RNA synthesis from the U_81 _of the template.

### TNTase activity of JEV NS5 protein

**Figure 9:**
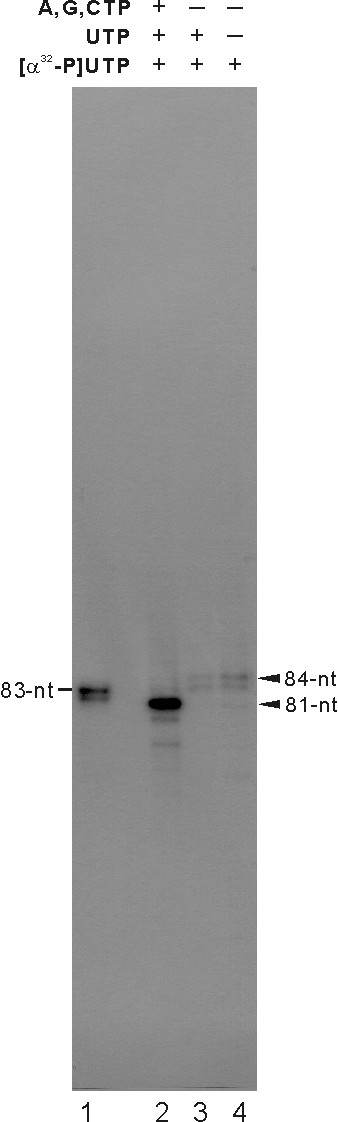
**TNTase activity of JEV NS5**. The 83-nt RNA was used as a template for RdRp and TNTase activity assays. For TNTase activity assays, reactions were performed in the presence of cold UTP and [α-^32^P] UTP (lane 3), or in the presence of single [α-^32^P] UTP (lane 4). An RNA product from the standard RdRp reaction mixture is shown as a control (lane 2). Lane 1, 5'-end labeled 83-nt RNA size marker.

Recent studies have shown that recombinant HCV NS5B and BVDV NS5B RdRps expressed in *E. coli *have an intrinsic TNTase activity; TNTase activity was not an activity of any cellular enzyme co-purified with the recombinant RdRps but an inherent function of the RdRps [24]. We set out similar TNTase assay conditions as depicted in Fig. 9. Similar to the previous results shown with HCV and BVDV RdRps, labeled products were detected in the TNTase activity assays performed in the presence of [α-^32^P] UTP and cold UTP (10 μM) or in the presence of [α-^32^P] UTP as a sole ribonucleotide substrate. Under these conditions, recombinant JEV NS5 synthesized template size and bigger-than-the template products but not the internally initiated 81-nt RNA product (Fig. 9, lanes 3 and 4). Consistently, in the standard RdRp reaction condition, JEV NS5 produced the 81-nt internally initiated RNA product (lane 2). Upon longer exposure to detect the TNTase activity in the presence cold UTP, we observed an additional minor product (<5 nt smaller than the 81-nt RNA product), which we were not able to detect under routine RdRp product analysis conditions. These results suggest that JEV NS5 might possess a TNTase activity, depending on reaction conditions.

## Discussion

In this study, we established an *in vitro *JEV RdRp assay system with a functional full-length JEV NS5 protein and characterized the mechanism of initiation of RNA synthesis. Results from this study showed that the recombinant JEV NS5 exhibits both RdRp and TNTase activities. The enzyme was capable of synthesizing RNA by a primer-dependent manner with a homopolymeric RNA template poly(A) and by *de novo *initiation with the RNA templates derived from JEV genome. Several reports showed that the TNTase activity is a common intrinsic property of many RdRps of *Flaviviridae*. The recombinant RdRps of HCV and BVDV have been shown to possess a TNTase activity under certain reaction conditions [24,25]. CSFV NS5B protein also was shown to possess TNTase activity, which added a single nucleotide to the 3' end of the 3'-UTR RNA template [26]. The JEV NS5 exhibited a weak but detectable level of intrinsic TNTase activity only when substrate ribonucleotides were limited (Fig. 9, lanes 3 and 4). Because the TNTase activity was detected only when limiting amount of cold UTP was added to the reaction mixture, its *in vivo *function, if any, remains unclear.

The JEV NS5 has a strong preference for Mn^2+ ^over Mg^2+ ^ion for RNA synthesis (Figs. 3 and 4). Considering that the intracellular concentration of Mn^2+ ^is much lower than Mg^2+ ^[11], the stringent Mn^2+^-dependent RdRp activity of JEV NS5 may not allow efficient JEV replication *in vivo*. Alternatively, this Mn^2+^-dependent RdRp activity might be simply an artifact of the unnatural *in vitro *conditions. It is also possible that this might be due to conformational difference of the recombinant JEV NS5 from the one in the virus-infected cells, in which JEV NS5 was likely to be membrane associated or to form a complex with other viral and/or cellular proteins. Nevertheless, similar stringent Mn^2+^-denpedent RNA synthesis using the homopolymeric RNA template poly(C) has been demonstrated with recombinant RdRps DEN and WNV [27]. Furthermore, when a DEN minigenome consisting of the 5'-end RNA (224 nt) and the 3'-end RNA (492 nt) of the viral genome was used as a template, the DEN RdRp activity was supported either by Mg^2+ ^or Mn^2+^, with the latter cation being a better cofactor. Our result is however contradictory to that obtained previously by *in vitro *RdRp assay using the JEV replication complex (RC) isolated from infected cells [28]. The RC displayed a strict dependence on Mg^2+ ^with absolutely no RdRp products being synthesized from the endogenous viral RNAs associated with the RC in the absence of Mg^2+^. The requirement of Mg^2+ ^for RNA synthesis by JEV RC might be due, in part, to its role as a cofactor for other viral and/or cellular proteins associated with the viral RNA polymerase.

During the course of flavivirus replication, minus-strand RNA is synthesized from the plus-strand genomic RNA. This intermediate form of RNA serves as a template for the synthesis of plus-strand genomic RNA that is synthesized at 10- to 100-fold higher levels than the minus-strand RNA [4]. Results from our *in vitro *RdRp assays using the 3'(+)UTR and 3'(-)UTR templates also supported the notion that a greater amount of plus-strand RNA than minus-strand RNA is synthesized in JEV- and DEN-infected cells [28,29]. The ratio of plus-to-minus strand RNA in the cells infected with these viruses was approximately 10:1. As shown in Fig. 5C, we demonstrated that the 3'(-)UTR served as a better template than the 3'(+)UTR for JEV NS5. This *in vitro *result also agreed with the result showing that the HCV NS5B RNA polymerase produces RdRp products more efficiently using the 3'(-)UTR than the 3'(+)UTR of the plus-strand viral RNA [30]. Such preference for plus-strand 3'-UTR over minus-strand 3'-UTR for RNA synthesis initiation might be correlated with the binding activity of RdRps to these *cis*-acting RNA elements. Indeed, a recent report showed that CSFV NS5B protein, a viral RdRp, recognizes the 3'(-)UTR more efficiently than the 3' (+)UTR [26]. Regarding the mechanism of initiation of RNA synthesis, JEV NS5 appears to be able to initiate RNA synthesis either *de novo *or by intramolecular priming *in vitro *using the viral genome-derived RNA templates tested in this work. From the 3'(+)UTR, JEVNS5 produced template size RNA as a major RdRp product, whereas bigger-than-template products were synthesized from the 3'(-)UTR RNA template (Fig. 5C). The nature of this bigger-than-template product is not clear at the present time, although it might be generated by extension of the 3'-end of the template by a snap-back priming mechanism or by extension of the newly synthesized nascent RNA products. The long single-stranded region at the 3'-end of the predicted secondary structure of the 3'(-)UTR might allow intramolecular priming.

Both nucleotide sequence and structure of 3'-UTR of plus-strand RNA viruses contain a *cis*-acting signal essential for the initiation of viral RNA replication. Although the size and sequence of the 3'-UTR vary among different flaviviruses, its secondary structure comprising stable stem-loops is predicted to be highly conserved. The last 80 to 90 nts at the 3'-end of various flavivirus genomes have been predicted to form stable stem-loop structures [31-33]. The last 83-nts of the 3'-UTR of JEV are predicted to form a stable stem-loop structure (Fig. 7C), which is similar to that formed with the 3'-end of the KUN genome [34]. The ability of JEV NS5 to use the 83-nt RNA template as a minimal template allowed us to explore its role in RNA synthesis. Our results revealed that JEV NS5 initiates RNA synthesis from an internal nucleotide U_81_, the third nucleotide from the 3'-end of the template (Fig. 7). We also observed that, although it is a very minor one, wild-type 83-nt RNA (83 WT), 83(Δ1), and 83(+U) generated an RNA product of 83-nt (Fig. 8). Because *de novo *initiated products from the 3'-end of these templates would generate 82, 83, and 84-nt product from 83(Δ1), 83 WT, and 83(+U), respectively, this product appears to be the 3'-end extended nascent RNA. Internal initiation of RNA synthesis was observed similarly with other *Flaviviridae *RdRps. For example, the penultimate cytidine at the 3'-end of KUN plus- and minus-strand RNA was shown to be essential for KUN RNA replication [34]. In addition, Oh *et al*. [17] and Kim *et al*. [35] showed that RNA synthesis initiates from an internal region of the 98-nt X-RNA template at the 3'-end of HCV genome, which is similar in terms of the RNA synthesis initiation mechanism to the result shown with JEV NS5 in this study. Because initiation of RNA synthesis from an internal nucleotide during viral RNA replication will result in loss of 3'-end genetic information, cellular and/or viral factors may play a role in the initiation of RNA synthesis from the 3'-end of genome. Previously, Chen *et al*. (8) showed that both JEV NS3 helicase and NS5 bind to the 3'-UTR of JEV genomic RNA. Moreover, the 36-kDa Mov34 protein was known to bind the 83-nucleotide 3' stem-loop structure recognized by the NS5 protein [23,36]. Those known viral and cellular proteins interacting with the 3'-end minimal *cis*-acting elements as well as other unknown factors might allow for JEV NS5 protein to initiate RNA synthesis from the 3'-end. The recombinant JEV NS5 protein will permit evaluating the effect of such *trans *factors on initiation of RNA synthesis.

Previous studies proposed that the 5' and 3' ends of the flavivirus RNA genome are able to interact directly between the cyclization sequence within the 3'-UTR and its complementary sequence in the capsid coding region following the 5'-UTR of the viral genome [37]. The cross-talk between these conserved *cis*-acting RNA elements of various flaviviruses was shown to be required for viral replication [38-40]. *In vitro *RdRp assays using DEN-infected cell lysates and recombinant viral RdRp from WNV showed that minus-strand RNA synthesis requires the interaction between the two terminal regions on the plus-strand viral RNA template through a cyclization motif, [18,21,41]. These previous results proposed that RNA synthesis from the 3'-UTR of plus-strand RNA requires the 5'-terminal region of the viral genome supplied in *cis *or *trans *so that cyclization of the 5'- and 3'-terminal regions forms a pan-handle-like structure. In contrast to *in vitro *RdRp assay results obtained with the above described flavivirus RdRps, our results demonstrated that JEV NS5 protein is capable of using the JEV 3'(+)UTR and the 83-nt RNA as templates (Fig. 5). RNA synthesis from these templates did not require the 5'-terminal cyclization motif in *cis *or *trans*. This result suggests that the sequence and/or structure of the 3'(+)UTR and the 83-nt RNA is sufficient for the NS5 to recognize the template and to initiate RNA synthesis, and indicates that cyclization of the JEV genome is not required for RNA synthesis *in vitro*. Thus, it is tempting to speculate that cyclization of the JEV RNA genome *in vivo*, if any, via direct RNA-RNA interaction or indirect interaction through other cellular and/or viral proteins, is involved in viral genome translation.

## Conclusion

We established an *in vitro *JEV RdRp assay system with an enzymatically active recombinant JEV NS5 protein. This recombinant JEV alone was able to recognize the *cis*-acting elements on both plus- and minus-strand 3'-ends. Like some of other flavivirus RdRps, it carries both RdRp and intrinsic TNTase activities. Its internal *do novo *RNA initiation from the 83-nt RNA template suggests that JEV RNA replicase complex might require viral and/or cellular proteins to direct RNA synthesis initiation form the 3'-end *in vivo*. The recombinant JEV NS5 protein will facilitate the analysis of the roles of such factors in initiation of RNA synthesis. The functional NS5 protein will also be useful for the development of target-specific inhibitors of JEV replication.

## Methods

### Cells and virus

Baby hamster kidney cells (BHK-21) were grown at 37°C in minimum essential medium (MEM, Invitrogen Life Technologies) supplemented with 5% fetal bovine serum (FBS, Invitrogen) and 1% penicillin/streptomycin sulfate (Invitrogen). The Nakayama strain of JEV, which was prepared from virus-infected mouse brain, was provided by the Department of Viral Disease, Korea National Institute of Health and used in our study. BHK-21 cells were infected with JEV at an multiplicity of infection of five as described previously [42].

### Construction of the recombinant JEV NS5 expression vector

To obtain a cDNA fragment encoding JEV NS5, viral genomic RNA from culture supernatants of JEV-infected BHK-21 cells was extracted with Trizol LS reagent (Invitrogen Life Technologies). After phenol/chloroform extraction, purified RNA was precipitated with isopropanol, washed once with 70% ethanol, and dissolved in RNase-free water. The RNA was reverse transcribed using Superscript II reverse transcriptase (Gibco-BRL) and the reverse primer 5'-GGGGTACCGATGACCCTGTCTTCCTG-3' as instructed by the manufacturer. The NS5 coding sequence was amplified by PCR, using Vent DNA polymerase (New England Biolabs) along with the forward primer (5'-CTAGCTAGCGGAAGGCCCGGGGGCAGG-3') and the reverse primer. The PCR product was digested with *Nhe*I and *Kpn*I and ligated into a similarly digested pTrcHisB (Invitrogen) vector to make the plasmid pTrcHisB-JEVNS5. The JEV NS5 mutant NS5_D668A _with a substitution of the first Asp with Ala in the GDD motif was generated by site-directed mutagenesis with two sequential rounds of PCR using oligonucleotides containing the target mutation as described previously [12]. The presence of the desired mutation was verified by DNA sequencing.

### Expression and purification of recombinant JEV NS5 protein from *E. coli*

JEV NS5 protein was expressed in *E. coli *TOP10 cells (Invitrogen) transformed with pTrcHisB-JEVNS5. The transformed cells were grown in LB medium containing 100 μg of ampicillin per ml to an optical density at 600 nm of 0.6–0.8 at 37°C, and protein expression was induced at 18°C for 12 h by addition of 1 mM isopropyl-β-D-thiogalactopyranoside (IPTG). The NS5 protein was purified by metal affinity chromatography using Ni-nitrilotriacetic acid (NTA)-agarose (Qiagen) resin as described previously [12]. The bound JEV NS5 proteins were step-eluted with the binding buffer containing 50 to 500 mM imidazole. JEV NS5-containing fractions were collected, dialyzed against a gel filtration column buffer (50 mM Tris-HCl [pH 7.8], 150 mM NaCl, 1 mM DTT, 0.5 mM EDTA), and then applied to a Sephacryl S-200HR column (Amersham Biosciences) at a flow rate of 0.5 ml/min. JEV NS5-containing fractions were collected and dialyzed against buffer A (50 mM Tris-HCl [pH 8.0], 50 mM NaCl, 1 mM DTT, 10% glycerol), and purified further by applying to an SP-Sepharose column (Amersham Biosciences). Adsorbed proteins were eluted with a 5 ml linear gradient of NaCl from 0.1 to 1 M in buffer A. Small aliquots of the NS5-containing fractions were stored at -80°C after dialyzing against buffer A. Protein concentrations were determined using a Bio-Rad protein assay kit with bovine serum albumin as a standard.

### RNA template preparation

The DNA templates for 3'(+)UTR (representing the 3'-UTR of JEV genome), 3'(-)UTR RNA (representing the region complementary to the 5'-UTR of JEV genome), the 83-nt RNA template (representing the 83-nt RNA from the 3'-end of JEV genome) were obtained by PCR with Vent DNA polymerase and the specific primers for 3'(+)UTR RNA (5'-TAATACGACTCACTATAG***CTAGTGTGATTTAAAGTA***-3' and 5'-***AGATCCTGTGTTCTTCC***-3'), 3'(-)UTR RNA (5'-***AGAAGTTTATCTGTGTG***-3' and 5'-TAATACGACTCACTATA***GGTTATCTTCCGTTCTAA***-3'), and the 83-nt RNA (5'-TAATACGACTCACTATA*GATCTTCTGCTCTATCTC*-3' and 5'-***AGATCCTGTGTTCTTCC***-3'). The T7 RNA polymerase promoter sequence is underlined, and the sequence complementary to the JEV genome sequence is shown in boldface and italic. pBAC^SP6^/JVFL/*Xba*I [43], which was kindly provided by Dr. Y. M. Lee at Chungbuk National University, Cheongju, Korea, was used as a template for PCR. Various DNA templates for derivatives of the 83-nt RNA were generated by PCR using a set of specific primers. The PCR-amplified DNA products were gel purified and used for *in vitro *transcription using T7 RNA polymerase as described previously [17]. RNA concentrations were estimated by measuring the absorbance at 260 nm.

### Enzymatic assays and analysis of products

RdRp assays were performed with 500 ng of purified JEV NS5 RdRp in a total volume of 25 μl containing 50 mM Tris-HCl (pH 8.0), 50 mM NaCl, 25 mM potassium glutamate, 1 mM MgCl_2_, 2.5 mM MnCl_2_, 1 mM DTT, 10% glycerol, 20 units of RNase inhibitor (Promega), cold ribonucleotide mixture (0.5 mM each ATP, CTP, and GTP, and 5 μM UTP), and 10 μCi of [α-^32^P] UTP (3,000 Ci/mmol; Amersham Biosciences). The reaction mixture was incubated with 200 ng of RNA template for 2 hr at 30°C. For RdRp reaction with poly(A) template, 1 μg of RNA was added to the reaction mixture as described above, except that 10 μM UTP was included in the reaction with 2 μCi of [α-^32^P] UTP. The RdRp reactions were performed in the absence or presence of 10 pmol of oligonucleotide (U)_20. _For the TNTase assay, 10 μCi of [α-^32^P] UTP mixed with or without 10 μM of cold UTP was used as a single ribonucleotide in the RdRp reaction mixture with the 83-nt RNA as a template. [γ-^32^P]-ATP incorporation assay was performed as described above for the standard RdRp assay, except that the reaction mixture contained cold ribonucleotide mixture (0.5 μM each CTP, GTP, and UTP, and 1 μM or 5 μM ATP) and 0.33 μM [γ-^32^P]-ATP (6,000 Ci/mmol, 10 mCi/ml; Amersham Biosciences).

RdRp reaction products were precipitated and resuspended in a denaturing loading buffer as described previously [12]. After heat denaturation and quick chilling on ice, the RNA products were resolved on 8 M urea-5% polyacrylamide gels (20 × 20 cm), unless otherwise specified. For size mapping, RNA products were separated in 8 M urea-6% denaturing polyacrylamide gels (20 × 45 cm). The gels were stained with ethidium bromide, photographed to locate the template positions, and then dried. The dried gels were exposed to X-ray film (BioMax XAR, Kodak) for autoradiography. The amounts of ^32^P-UMP incorporated into the products were measured in a LS-6500 multi-purpose scintillation counter (Beckman).

### Nuclease S1 treatment

RdRp products were digested with 4 units of nuclease S1 per ml (Promega) at 37°C for 30 min in nuclease S1 digestion buffer (30 mM NaOAc [pH 4.6], 1 mM ZnSO_4_, 5% glycerol) containing either a low (50 mM) or high (500 mM) concentration of NaCl.

### Preparation of RNA size markers

For 5'-end ^32^P-labeling of the 83-nt RNA, *in vitro *transcripts were dephosphorylated with calf intestine alkaline phosphatase (Takara Bio Inc.), and then phosphorylated with T4 polynucleotide kinase (New England Biolabs) in a final reaction volume of 50 μl containing 50 μCi [γ-^32^P] ATP and 0.1 mM cold ATP. After incubation at 37°C for 30 min, free nucleotides were removed using a Sephadex G-25 spin column, and the labeled RNA was purified from the denaturing polyacrylamide gel as described previously [17]. The ^32^P-labeled 83-nt RNA was partially digested by incubation in Na_2_CO_3_/NaHCO_3 _buffer (pH 10) for 10 min at 90°C.

### Prediction of RNA secondary structure

RNA secondary structures were analyzed by the Mfold program [44]. The structure drawings were edited and annotated using the RnaVis 2 program [45].

### SDS-PAGE, Western blot analysis, and MALDI-TOF analysis

For Western blot analysis, protein samples were resolved by sodium dodecyl sulfate-polyacrylamide gel electrophoresis (SDS-PAGE), and then transferred to nitrocellulose membranes (Amersham Biosciences). The samples were blocked using 5% skim milk in TBST (20 mM Tris-HCl [pH 7.4], 150 mM NaCl, 0.1% Tween 20), then reacted with an anti-His antibody (Qiagen) at a 1:1,000 dilution, followed by incubation of goat anti-mouse lgG conjugated with peroxidase (Sigma-Aldrich). The immunoblots were washed and then immunoreactive protein bands were visualized using enhanced chemiluminescence (ECL, Amersham Biosciences). Silver staining of gels and matrix-assisted laser desorption/ionization-time of flight mass spectrometry (MALDI-TOF MS) analysis were performed as described previously [46].

## Authors' contributions

YGK, JSY, and CMK purified JEV NS5 protein and characterized its biochemical properties. JHK made the original clone of JEV NS5. YGK and JSY prepared the initial draft of the manuscript. JWO conceived of the study, coordinated all the components of the project, and prepared the final manuscript. All authors have read and approved the final manuscript.
